# N-terminal basic amino acid residues of *Beet black scorch virus* capsid protein play a critical role in virion assembly and systemic movement

**DOI:** 10.1186/1743-422X-10-200

**Published:** 2013-06-20

**Authors:** Xiaofeng Zhang, Xiaofei Zhao, Yanjing Zhang, Shaofang Niu, Feng Qu, Yongliang Zhang, Chenggui Han, Jialin Yu, Dawei Li

**Affiliations:** 1 State Key Laboratory of Agro-Biotechnology and Ministry of Agriculture Key Laboratory of Soil Microbiology, College of Biological Sciences, China Agricultural University, Beijing 100193, China; 2 Department of Plant Pathology, Ohio Agricultural Research and Development Center, The Ohio State University, Wooster OH 44691, USA

**Keywords:** *Beet black scorch virus*, Capsid protein, RNA binding, Virion assembly, Viral systemic movement

## Abstract

**Background:**

*Beet black scorch virus* (BBSV) is a small single-stranded, positive-sense RNA plant virus belonging to the genus *Necrovirus*, family *Tombusviridae*. Its capsid protein (CP) contains a 13 amino acid long basic region at the N-terminus, rich in arginine and lysine residues, which is thought to interact with viral RNA to initiate virion assembly.

**Results:**

In the current study, a series of BBSV mutants containing amino acid substitutions as well as deletions within the N-terminal region were generated and examined for their effects on viral RNA replication, virion assembly, and long distance spread in protoplasts and whole host plants of BBSV. The RNA-binding activities of the mutated CPs were also evaluated *in vitro*. These experiments allowed us to identify two key basic amino acid residues in this region that are responsible for initiating virus assembly through RNA-binding. Proper assembly of BBSV particles is in turn needed for efficient viral systemic movement.

**Conclusions:**

We have identified two basic amino acid residues near the N-terminus of the BBSV CP that bind viral RNA with high affinity to initiate virion assembly. We further provide evidence showing that systemic spread of BBSV in infected plants requires intact virions. This study represents the first in-depth investigation of the role of basic amino acid residues within the N-terminus of a necroviral CP.

## Background

The capsid proteins (CPs) encoded by viruses play important roles at various stages of viral multiplication cycles [1]. One of the primary functions of CPs is to form virus particles that protect the corresponding viral genomes from degradation by nucleases in the infected host cells, and ensure the successful transmission of viruses between hosts. During the assembly of RNA virus particles, CPs also act as RNA chaperones to fold the viral genomic RNA into a structure compatible with the proper symmetry of the mature particles [2]. The interaction between CP and genomic RNA plays a key role in ensuring the specificity of virion assembly and the stability of assembled virions [3,4]. In general, a basic region is present in the N or C terminus of the CP of many positive sense (+) RNA viruses that is responsible for interacting with viral RNA with high affinity [5-13].

The genus *Necrovirus* of Family *Tombusviridae* encompasses some of the smallest (+) RNA viruses with monopartite genomes and icosahedral virion symmetry. Among them, the Toyama isolate of *Tobacco necrosis virus* (TNV) was the first necrovirus for which the crystal structure of assembled virions was determined [14]. Analysis of TNV virion structure identified an N-terminal basic region in its CP that is disordered and oriented toward the inside of the particle [14]. Similar basic regions were also found in the CPs of other necroviruses, including TNV-D [15], *Olive latent virus 1* (OLV-1) [16], TNV-A [17,18], and *Beet black scorch virus* (BBSV) [19]. However, the functional role of basic amino acid residues in this region is still poorly understood.

Most plant viruses also require CP for their long distance spread in the infected plants. Indeed, a number of viruses have been found to access long distance movement in the form of virions [20-24]. However, since CPs could accompany viral genomes either as CP-RNA complexes or as assembled virions, it is unclear whether assembled virions are required for the systemic movement of these viruses. The limited analysis of necrovirus CPs suggested that either CP alone or virions could facilitate their long distance movement [25,26].

**Figure 1:**
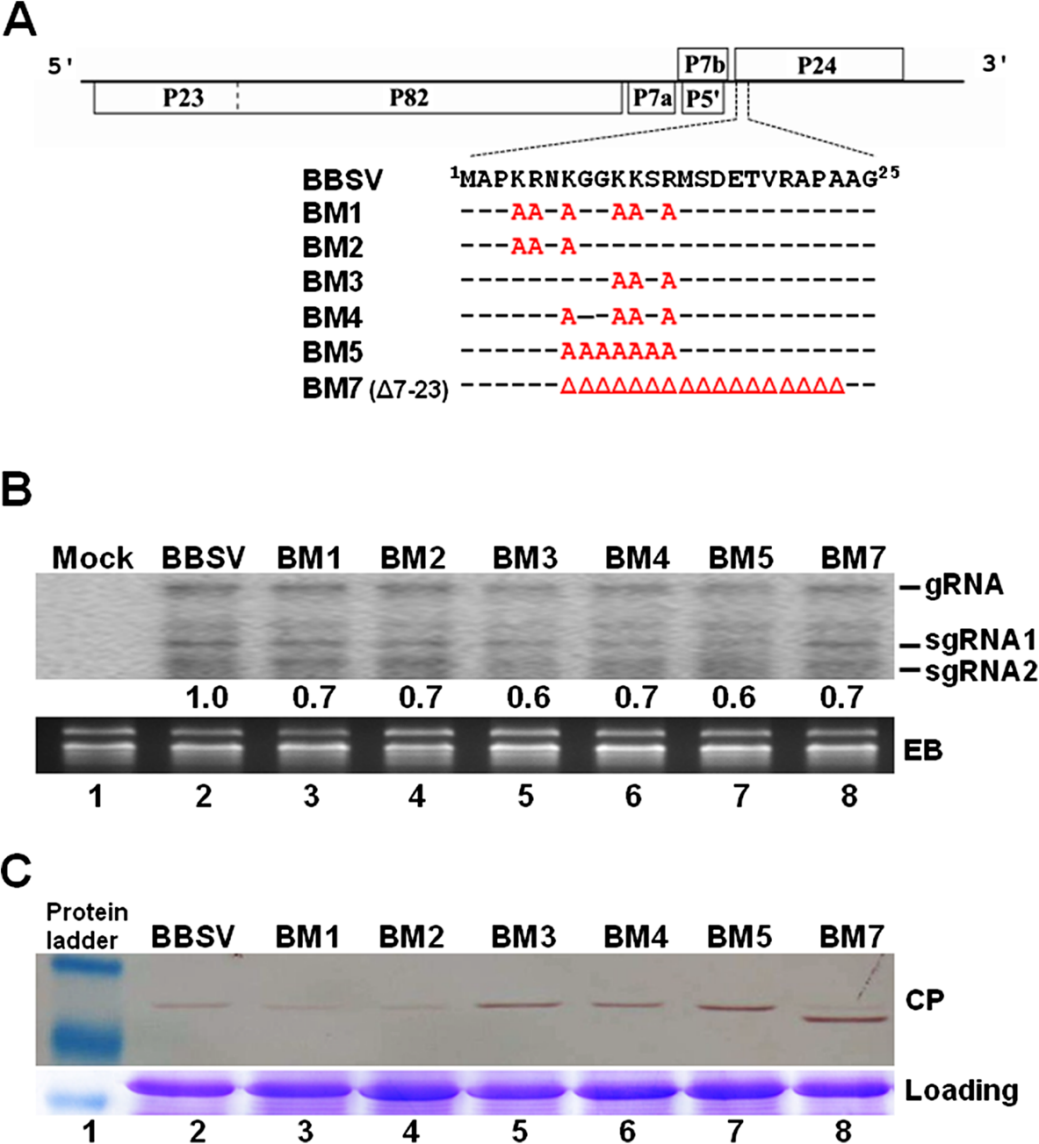
**Replication of BBSV and its mutants in *N. benthamiana *protoplasts.** (**A**) Schematic representation of BBSV and the six mutants containing changes within the N-terminus of CP. P23 and its translational readthrough product, P82, are needed for viral replication. P7a, P7b and P5’, three small proteins encoded by the middle portion of BBSV genome, are required for viral cell-to-cell movement. P24 encoded by the 3’ portion of the genome is the viral coat protein (CP). Amino acid residues changed in the CP mutants (BM1-5, BM7) are highlighted in red. (**B**) Northern blot analysis of viral RNAs accumulated in *N. benthamiana* protoplasts. Bands corresponding to viral genomic (g) RNA and subgenomic (sg) RNAs (sgRNA1 and 2) are indicated on the right. (**C**) Western blot detection of WT as well as mutant BBSV CPs in transfected protoplasts.

BBSV is a single-stranded (+) RNA virus of the genus *Necrovirus*. It was first identified in sugar beets grown in China [27,28], and subsequently reported in North America, Europe, and Middle East [29-31]. Its 28 nm icosahedral particle encapsidates one 3,644 nucleotide (nt) monopartite genome that encodes six open reading frames (ORFs; Figure 1). The 5’-proximal 23 kilodalton (kDa) protein (P23) and its read-through product (P82) are required for the replication of the genomic RNA (gRNA), as well as the synthesis of two subgenomic RNAs (sgRNAs), the latter serving as mRNAs for the translation of other four ORFs [32]. The three small overlapping ORFs located in the central region of the genome, P7a, P7b and P5’, were identified as movement proteins (MPs) [32,33]. The 3’-proximal ORF encodes the 24 kDa viral CP. Recent studies found that BBSV CP and MP could also enter the nuclei of the infected cells and thereby contribute to the elicitation of various leaf symptoms [19,27,32-34]. In addition, BBSV CP was found to be dispensable for viral cell-to-cell movement, but essential for the development of BBSV-specific systemic symptoms in the host *Nicotiana benthamiana*[32].

In the current report, we document a systematic analysis of the N-terminal basic region of BBSV CP through the creation of a series of deletion and single amino acid substitution mutants within this region. The resulting mutants were exhaustively examined in protoplasts, local, and systemic leaves of *N. benthamiana* for their competence in replication, particle assembly, as well as systemic spread. The mutated CPs were also examined *in vitro* for their RNA-binding capacities. We conclude from these experiments that the lysine (K) and arginine (R) residues at the amino acid positions 4 and 5 of BBSV CP are essential for efficient CP-RNA interactions, and that intact virions are required for viral systemic spread.

## Results

### Replication of BBSV RNA was not appreciably compromised by the set of mutations created in this study

In a previous study, we have identified a motif rich in K and R residues, ^4^KRNKGGKKSR^13^, in the N terminus of BBSV CP that mediated the nuclear localization of CP, leading to the speculation that this K/R-rich motif could play an important role in BBSV long distance movement and symptom development [19]. To further assess the function of basic amino acid residues in this motif, a series of mutants were generated by either replacing one or more basic amino acids with alanine (A) or deleting the complete motif (Figure 1A). Specifically, BM1 contained six amino acid substitutions that replaced all six basic residues with A, whereas BM2 and BM3 contained the first and the last three of the six substitutions in BM1, respectively. Additionally, BM4 contained the last four of the BM1 substitutions, whereas BM5 changed all amino acids from positions 7 to 13 to A. Finally, BM7 is a deletion mutant containing a 17 amino acids deletion that spanned from positions 7 to 23. To first determine whether these changes compromised the replication of BBSV gRNA, we subjected these mutants to single cell infections in *N. benthamiana* protoplasts. As shown in Figure 1B, all of the mutants accumulated both gRNA and sgRNAs to levels similar to WT BBSV. Thus, the N-terminal basic region of BBSV CP does not appear to contribute to BBSV genome replication in *N. benthamiana* single cells.

We also examined the levels of BBSV CP in these protoplast cells. As shown in Figure 1C, all of the mutated forms of CP accumulated to detectable levels (Figure 1C, top panel, lanes 3 – 8). Notably, the accumulation of BM1 and BM2 CPs was consistently lower than either WT CP or other mutants in two separate experiments (Figure 1C, compare lanes 3 and 4 with other lanes; and data not shown). Since mutations in BM1 and BM2 did not involve changes in either 5’ or 3’ untranslated region of the CP sgRNA, we consider it unlikely that the lower levels of BM1 and BM2 CPs were caused by reduced CP translation. Rather, they more likely reflected the decreased stability of mutated CPs, which could in turn be caused by their inability to be incorporated into virus particles. Interestingly, BM1 and BM2 share two amino acid substitutions (^4^KR^5^ → AA) that are absent in other mutants, thus suggesting a critical role of these two amino acid residues in CP stability.

### The first two basic residues (^4^KR^5^) of BBSV CP are essential for the CP to bind BBSV genomic RNA *in vitro*

**Figure 2:**
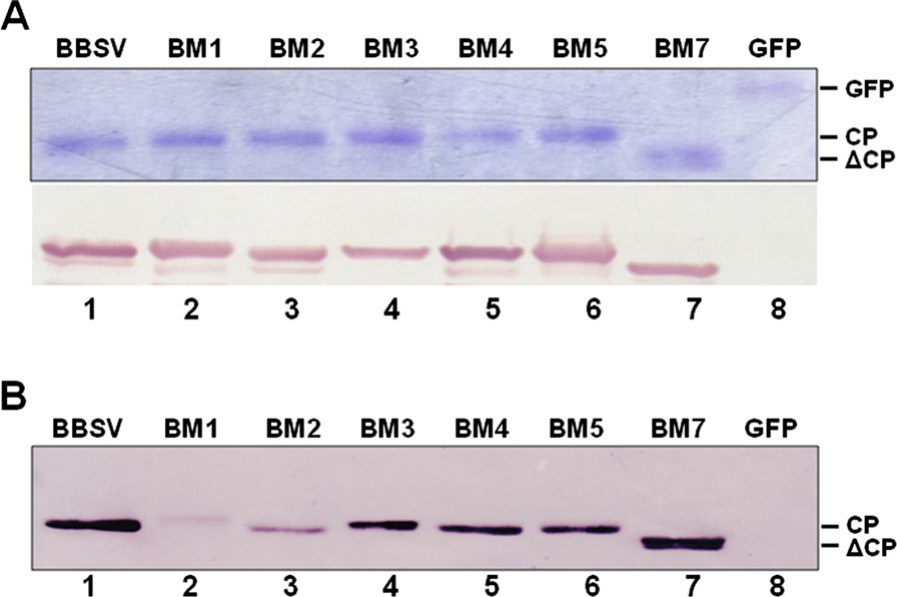
**Delineation of the amino acid residues within the N-terminus of BBSV CP involved in RNA binding with North-western blot analysis.** (**A**) Confirmation of the quantity and purity of wild type (WT) and mutated BBSV CPs prepared with an *E. coli* expression system using SDS–PAGE (top) and Western blot (bottom). GFP was used as a control. (**B**) RNA binding capability of WT and mutant CPs were assayed using non-radioactively labeled BBSV RNA (see Materials and Methods for details). The experiment was repeated three times with similar results.

The reduced stability of BM1 and BM2 CPs could be due to their inability to form stable virions, thus exposing the unassembled CPs to protein degradation processes in the infected cells. To test this possibility, we decided to assess the ability of various CP mutants to interact with BBSV viral RNA, which is considered to be the first step of virion assembly. The WT and mutant CPs were expressed and purified using an *E. coli* expression system. The yield of purified proteins was first confirmed by Coomassie blue-stained SDS-PAGE gels, as well as Western blot with BBSV CP antibody. As shown in Figure 2A, we were able to obtain WT as well as mutant CPs with satisfactory yields. An equal amount of purified WT and mutant BBSV CPs were then separated on an SDS-PAGE gel, transferred to nitrocellulose membrane, and renatured prior to being exposed to the digoxigenin-labeled BBSV genomic RNA, with the similarly purified GFP as a negative control.

Figure 2B shows the result of one of the representative North-western experiments. As shown in lane 8, The GFP protein failed to immobilize any BBSV RNA, thus confirming that the RNA-binding is a specific property of WT BBSV CP (lane 1). Significantly, the RNA-binding activity of BM3, 4, 5, and 7 were only slightly reduced (Figure 2B, lanes 4–7), thus revealing less significant participation of the basic residues after amino acid position 6 of BBSV CP (see Figure 1A for detailed changes in the respective mutants). It is particularly noteworthy that BM5 CP (Figure 2B, lane 6), despite the replacement of all amino acid residues between positions 7 and 13 with A, was still able to bind BBSV RNA with affinity levels approaching that of WT CP. By contrast, BM1 and BM2 CPs, which shared the ^4^KR^5^ → AA changes, were able to bind only trace amounts of BBSV RNA (Figure 2B, lanes 2 and 3). Together, these results clearly illustrated that these two basic residues are indispensible for the RNA-binding activity of BBSV CP. Therefore, the reduced accumulation of BM1 and BM2 CPs in infected protoplasts, in which these two residues were replaced by alanines, was most likely caused by a lack of RNA-binding activity, and hence an inability to assemble stable virions.

### Virus assembly was compromised by the N-terminal mutations to varying degrees

**Figure 3:**
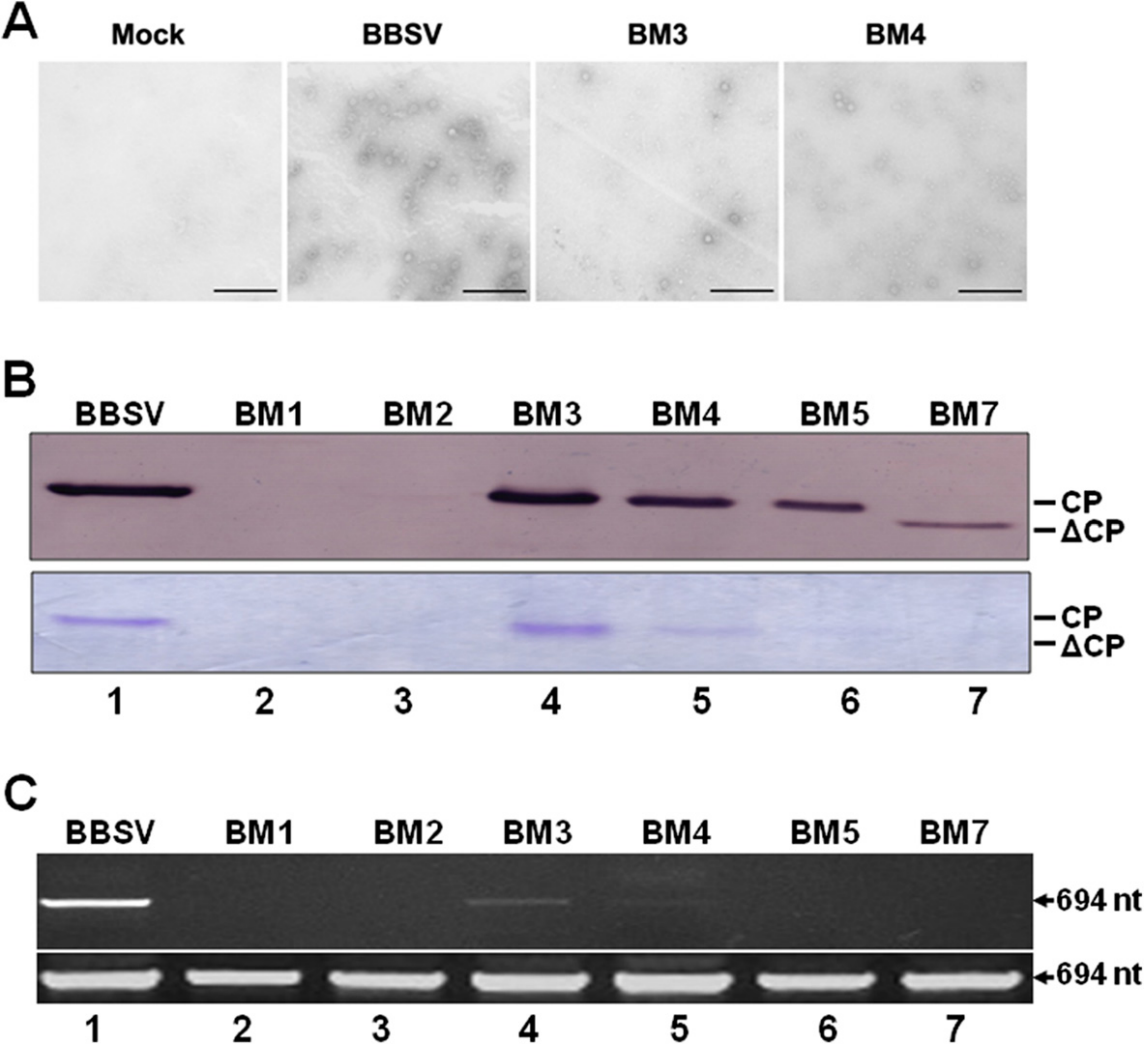
**Detection of stable virions isolated from *N. benthamiana *leaves inoculated with transcripts of various mutants.** (**A**) Electron micrographs depicting virions isolated from leaves inoculated with mock, WT BBSV, and BM3 and BM4 mutants. Bar = 200 nm. (**B**) Detection of mutated CP in the purified virions. Purified virions were separated on an SDS-PAGE gel and stained with Coomassie brilliant blue R250 staining (top panel), or subjected to Western blot analysis using a BBSV CP antibody to confirm the CP identity (bottom panel). (**C**) Detection of protected viral RNAs in virions underwent an incubation period that permitted the degradation of unprotected RNAs, with RT-PCR (top panel). The bottom panel shows the RT-PCR results with total RNA extracted from the same inoculated leaves (without 30-minute inoculation), serving as controls for viral RNAs available for virion assembly.

Having revealed the critical role of ^4^KR^5^ in the interaction between CP and viral RNA of BBSV, we next wanted to determine whether substituting these two amino acid residues indeed impaired BBSV virion assembly. We hence tried to purify virions from the inoculated leaves of the *N. benthamiana* plants infected with the mutant transcripts, at 10 days post inoculation (dpi. See Methods section for details). The purified virions were first examined with electron microscopy (EM). Surprisingly, only BM3 and BM4 produced virions at sufficiently high concentrations to permit the detection by EM (Figure 3A, right two panels). In an attempt to detect lower levels of virion assembly by other mutants, we then subjected the virion preparations to Western blot with a BBSV CP antibody. As shown in Figure 3B (top panel), a BBSV CP-specific band was detected in BM3, 4, 5, and 7 samples, with decreasing intensities, but not in BM1 and BM2 samples. It should be noted that the extensive purification procedure would have removed most other proteins not associated with assembled virions. Indeed, the Coomassie blue-stained PAGE gel (Figure 3B, bottom panel) also showed that the bands corresponding to the mutated CPs were the only ones present. These results suggested that the mutated CPs of BM3, 4, 5, and 7 were still able to form virions, albeit at reduced and varying efficiencies. By contrast, virion assembly by BM1 and BM2 mutants, if occurred at all, was below the detection limit of our procedure. These results strongly suggest that inability of BM1 and BM2 CPs to bind viral RNA prevented the mutated CPs from virion assembly. Conversely, RNA-binding by ^4^KR^5^ of BBSV CP likely serves as the initiation step of virion assembly.

### Virions formed by BM3, 4, 5, and 7 CPs were less capable of protecting BBSV genomic RNA

Data presented in Figure 3A and 3B suggest that, while ^4^KR^5^ are indispensible for virion formation, they may not be sufficient for WT level assembly as particles of BM3, 4, 5, and 7 mutants accumulated to lower levels than WT. To further elucidate the role of N-terminal basic residues at positions after ^4^KR^5^, we next examined whether the virions formed by BM3, 4, 5 and 7 CPs protected viral genomic RNAs to the same extent as WT CP. This was accomplished by comparing the levels of total BBSV RNAs in the infected cells with those protected by virions, using an RT-PCR procedure. The total amount of viral RNAs in the infected cells was estimated by RT-PCR amplification of a BBSV-specific fragment from RNA samples without RNase treatment. By contrast, to obtain BBSV RNAs protected by virions, the leaf tissues were homogenized to disrupt the cells, allowing for the release of both virions and cellular RNases. The homogenates were then incubated at 37°C for 30 minutes to facilitate the degradation of any unprotected RNA by plants’ own RNases. The viral RNAs protected by virions were then extracted using a standard procedure.

As shown in Figure 3C, while BBSV RNA levels in the leaves inoculated with all mutants were similar to (bottom panel, lanes 4 and 5), or only slightly lower (lanes 2, 3, 6 and 7) than those inoculated with WT BBSV (lane 1), as reflected by the levels of RT-PCR products derived from total RNA samples without RNase treatment, the levels of BBSV RNAs protected by mutant virions were drastically lower, being barely detectable for BM3 and BM4, and below the detection limit of our procedure for all other mutants (BM1, 2, 5, and 7). These results indicated that virions formed by BM3, 4, 5, and 7 were all partially defective in terms of protecting viral RNA from degradation, with some (BM5 and BM7) more severely than others (BM3 and BM4). Thus, while BM3 and BM4 CPs were capable of form virions with considerable efficiencies, the virions formed were substantially less competent at protecting the encapsidated viral RNA. Taken together, the data presented here demonstrated that all basic residues at the N-terminus of BBSV CP are important at ensuring proper assembly of BBSV virions. Nevertheless, ^4^KR^5^ are the two most critical basic residues responsible for initiating the assembly process through RNA-CP interaction.

### Behavior of BBSV mutants in whole plants is highly correlated with the ability of their CPs to form intact virions

**Figure 4:**
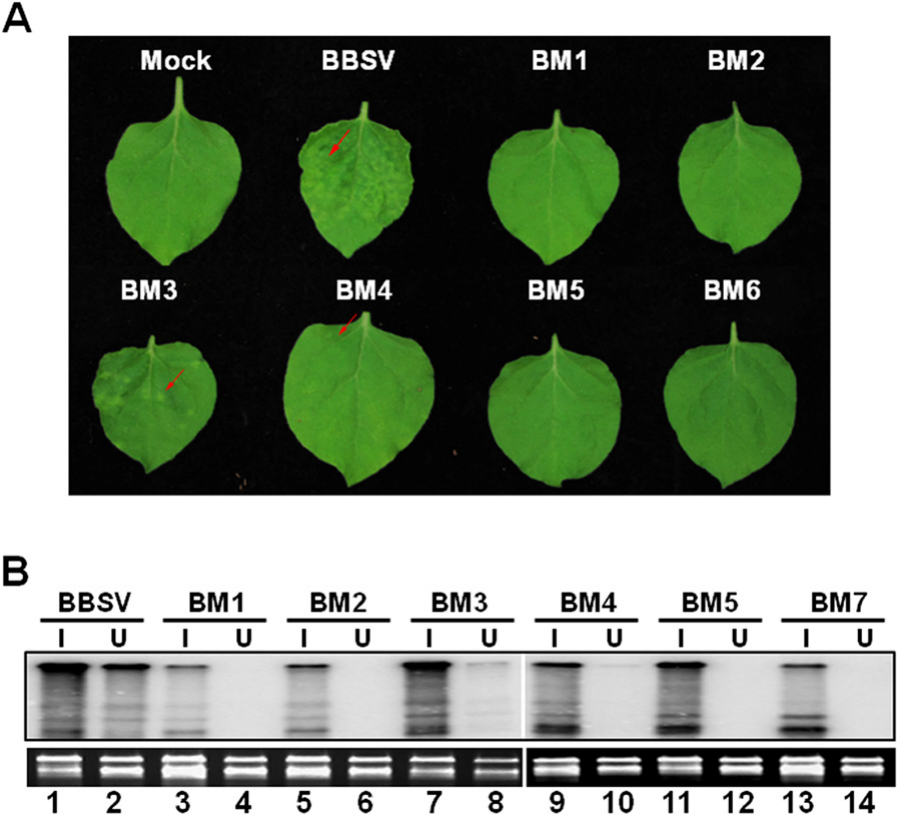
**Systemic symptoms of *N. benthamiana *plants inoculated with BBSV and its mutants, and detection of viral RNAs with Northern blot assay.** (**A**) Systemic symptoms of *N. benthamiana* plants inoculated with *in vitro*-synthesized RNAs of CP mutants of BBSV as observed at 15 dpi. (**B**) Detection of viral RNAs in both inoculated (I) and upper (U) leaves of *N. benthamiana* with Northern blot assay, using a specific probe complementary to BBSV 3’UTR.

Having revealed the varying capabilities of mutant CPs in assembling intact virions, we next assessed whether defects in virion assembly impede the systemic movement of these mutants in *N. benthamiana* plants. We hence used *in vitro* transcripts of the mutant constructs to inoculate young *N. benthamiana* plants and closely followed the infected plants for a four weeks period. Figure 4A depicts typical upper uninoculated leaves (UULs) of inoculated plants at 15 dpi. WT BBSV infections induced easily identifiable symptoms manifested as merged chlorotic patches on UULs (Figure 4A, top row, second leaf). By contrast, BM3 infections caused far fewer chlorotic patches on UULs (bottom row, first leaf). Even more dramatically, BM4 infections led to sporadic patches visible on only a few UULs (bottom row, second leaf). Finally, none of the other mutants (BM1, 2, 5 and 7) caused visible symptoms on UULs (Figure 4A).

To ensure the integrity of the mutant transcripts, and to verify the symptom differences described above, we then harvested both inoculated leaves and UULs from these plants, and extracted total RNA from the leaves, and subjected the RNA samples to Northern blot with a BBSV-specific probe. As shown in Figure 4B, both BBSV gRNA and sgRNAs were readily detected in the inoculated leaves exposed to all mutants (Figure 4B, the “I” lanes), including BM1 and BM2 which were unable to form virions (Figure 4B, lanes 3 and 5). These results suggest that virion assembly was not essential for the multiplication of mutant viruses in inoculated leaves. However, BBSV gRNA and sgRNAs were only detected in UULs of WT, BM3, and BM4-infected plants, with BM3 and BM4 at barely detectable levels (Figure 4B, compare lanes 2, 8, and 10). These results are consistent with the disease symptoms elicited by the corresponding mutants, suggesting that efficient systemic spread of BBSV is dependent on the assembly of intact or near intact virions capable of protecting viral RNAs.

Since Northern blot hybridization is a relatively low throughput procedure not suitable for analyses of large amounts of plants, we decided to use RT-PCR to examine additional plants and gauge the extent of systemic movement of other mutants not detected in UULs with the Northern blot procedure. The results summarized in Table 1 showed that, with more than 30 plants examined for each mutant, genomes of BM3 (31/40), BM4 (10/36), BM5 (3/36), and BM7 (1/36) were all detected in the UULs of at least one plant. By contrast, BM1 and BM2 were undetectable in the UULs of any of the inoculated plants. Strikingly, for the four mutants with varying degrees of virion assembly (BM3, 4, 5, and 7; see Figure 3), the extent of systemic spread was positively correlated with the level of virions detectable in inoculated leaves. These results lend further support to the conclusion that BBSV moves systemically in the form of assembled virions.

**Table 1:** **Systemic movement of BBSV mutants in *N. benthamiana***

**Inoculum**	**Symptoms**^**∆**^	**RT-PCR**
BBSV	30/30*	30/30**
BM1	0/32	0/32
BM2	0/32	0/32
BM3	27/40	31/40
BM4	8/36	10/36
BM5	1/36	3/36
BM7	1/32	1/32

Symptoms^∆^, merged chlorotic patches.

* Number of plants with symptoms/Number of plants inoculated with respective BBSV transcripts.

** Number of plants with mutant genomes detectable by RT-PCR/Number of plants inoculated with respective BBSV transcripts.

## Discussion

In this report, we have investigated an N-terminal K/R-rich motif of BBSV CP for its role in high affinity binding of viral RNA, virion assembly, protection of viral RNA, as well as viral systemic movement. A set of six mutants was generated, each altering this motif to a different extent. All of the mutants were able to replicate normally in single cells, thus ruling out a significant role for this motif in genome replication. However, while all mutants showed varying degrees of defect in terms of viral systemic movement and virion integrity, it is the BM1 and BM2 that showed the most severe debilitation in all four sets of testing. These experiments implicated the first two basic residues of BBSV CP (^4^KR^5^) in the initiation step of virion assembly through RNA-binding.

Previous studies using TNV showed that the N-terminal basic region of CP faces to the interior of the virion, is disordered, and likely interacts with viral RNA [14,35]. Our results showing the involvement of the first two basic residues in RNA-binding are consistent with these previous studies. Our results also clearly demonstrated that efficient virion assembly correlates with successful systemic dissemination of BBSV. The importance of particle assembly in viral long distance movement has been demonstrated with TNV-D^H^ and OLV-1, two other necroviruses [25]. Therefore, this may be a common feature for necroviruses.

Viral CPs has been found to be involved in the systemic movement of many plant viruses. While it is possible that the genome of some viruses could interact with CP to form nucleoprotein complex that traffics through the vascular tissues, most viruses have been found to require virion formation for systemic infection [20-22,26,36,37]. Our study, by showing that some mutants (e.g. BM7) could bind viral RNA with high affinity but were unable to move systemically with meaningful efficiency, adds additional support to this model.

We reported earlier that BBSV CP could localize to the nuclei of infected cells, and the N-terminal K/R-rich domain contains the corresponding nuclear localization signal [19]. In the present study, most mutants we constructed would be expected to have disrupted this signal yet they all replicated efficiently in *N. benthamiana* protoplasts, ruling out a prominent role for CP nuclear localization in viral genome replication. While our results clearly implicated this basic region in virion assembly and viral systemic movement, the current experimental system does not preclude an additional role of this motif in regulating virus-host interactions through the nuclear localization of BBSV CP [19].

An N-terminal domain rich in basic residues has been found in many other viruses including *Cucumber mosaic virus*, *Brome mosaic virus*, and *Cowpea chloric mottle virus*[38]. A common feature of such domains is that they play important roles in virion assembly and viral systemic movement but rarely affect viral genome replication in single cells. Our results are in line with these earlier observations. Nevertheless, our study provides additional novel insights by identifying two key basic residues at the N-terminus of BBSV CP that are indispensible for the binding of viral RNA, and simultaneously essential for virion assembly, thus establishing a direct connection between viral RNA-binding and initiation of virion assembly.

## Conclusions

We report a detailed characterization of the 13 amino acid long N-terminal basic region of BBSV CP. We identified two amino acid residues, ^4^KR^5^, that are specifically needed for binding of viral RNA by CP. We further showed that RNA-binding by these two residues is needed for the assembly of BBSV virions. Finally, we demonstrated that intact virions are the primary vehicle of BBSV systemic spread in *N. benthamiana* plants. This is the first report of precise delineation of specific amino acids in a necrovirus that plays an indispensible role in virion assembly.

## Methods

### Deletion and site-directed mutagenesis of BBSV CP

Overlapping-PCR with appropriate primers (sequences available upon request) was used to engineer both deletions of a desired group of amino acids, and substitutions of specific amino acids within BBSV CP (Figure 1). The mutated BBSV CPs were incorporated into the infectious BBSV cDNA clone (pUBF52) using the *Mfe*I and *Bam*HI, which cut the BBSV cDNA at unique sites flanking the CP ORF. The regions between the *Mfe*I and *Bam*HI sites were sequenced to confirm the presence of the mutations and to ensure that no unintended mutations were introduced.

### *In vitro* transcription

Prior to *in vitro* transcription, infectious clones containing wild-type (WT) and mutant cDNAs of BBSV were linearized with *Sma*I. The run-off transcription was carried out at 37°C for 1.5 hours with an *in vitro* transcription kit equipped with T7 RNA polymerase (Promega). The concentration of transcripts was estimated with UV spectrophotometry and gel electrophoresis.

### Infection of protoplasts and plants, and analysis of RNA and CP

Protoplasts were prepared from *N. benthamiana* leaves as previously described [39]. Approximately 10^6^ of freshly isolated protoplasts were transfected with 20 μg of *in vitro* transcripts with a PEG-calcium chloride-mediated transfection procedure. The transfected protoplasts were incubated for 18 hours before being subjected to total RNA extraction using the Trizol reagent (Invitrogen). The replication efficiency of various BBSV RNAs was evaluated with Northern blot hybridizations with BBSV-specific probes. For mechanical inoculation of intact plants, *in vitro* transcripts were diluted to 1 μg/μl with the inoculation buffer (50 mM glycine, 30 mM K_2_HPO_4_, 1% bentonite, 1% celite, pH 9.2), and rubbed gently onto young leaves of the plants to be tested. The inoculated plants were placed in a greenhouse room set at 18°C. At 7–8 dpi, total RNA and protein were extracted from both the inoculated and systemic leaves [32], and used for Northern blot and Western blot analyses. For Northern blotting, ^32^P-labeled riboprobe complementary to the 3’ terminal 300 nt of the BBSV genome was used to detect positive-stranded BBSV RNA progeny. For Western blotting, specific antibody to BBSV CP was used to detect the accumulation of BBSV CP [34].

### Particle purification and stability assay

Mechanically inoculated *N. benthamiana* leaves were used for particle purification and RNase-sensitivity analyses. BBSV particle purification was performed according to a previously published protocol with minor modifications [34]. Briefly, 30 grams (g) of inoculated leaves were collected and ground thoroughly in liquid nitrogen with a sterile mortar and pestle. Two volumes (w/v) of 0.2 M sodium phosphate buffer (pH 7.0) containing 0.2% (v/v) β-mercaptoethanol were added, and the mixture was incubated on ice with continuous shaking for approximately one hour. The homogenate was filtered and the sap was centrifuged at 8,000 rpm for 15 min in a Sorvall SS-34 centrifuge (Beckman Coulter, Fullerton, CA, USA) to remove the insoluble material. The supernatant was then transferred into a new sterile container, slowly mixed with prechilled chloroform to a final concentration of 15%-20% (v/v), and further stirred in an ice bath for 30 min. Next, the emulsified solution was centrifuged at 12,000 rpm for 15 min at 4°C. The clarified supernatant was collected and mixed with 1/10 volume of 30% PEG_6,000_, 10% NaCl. After being incubated at 4°C with stirring for 2 h, the mixture was centrifuged at 13,000 rpm for 20 min at 4C. The pellet was resuspended with 0.2 M sodium phosphate buffer (pH 7.0) and centrifuged at 14,000 rpm for 90 min at 4°C. Virus particles in the clarified supernatant were sedimented by ultracentrifugation through a 5 ml 20% sucrose cushion using a Beckman Type 70Ti rotor (35,000 g, 60 min, 4°C). The pellet was resuspended with 1 ml ddH_2_O. The purified virions were used for Western blotting analysis.

The stability of virus particles formed by mutant CPs was assayed using an RNase-sensitivity assay as previously reported [40]. 0.2 g inoculated leaves were ground in liquid nitrogen with 200 μl PIPES buffer (50 mM PIPES, pH 6.5, 0.1% Tween) and incubated at 37°C for 30 min to permit degradation of viral as well as cellular RNAs by endogenous RNases. The RNase-treated virion extract was then subjected to isolation of RNA using Trizol (Invitrogen). The amount of remaining viral RNA was determined by RT-PCR amplification of a 965 bp BBSV-specific fragment.

### Bacterial expression and purification of WT and mutant BBSV CPs

cDNAs of WT and mutant BBSV CPs were cloned into the pGEX-KG vector [41] using *Bam*HI and *Sal*I to acquire GST fusion proteins. The derived constructs were transformed into *Escherichia coli* BL21(DE3) pLysS cells. 0.2 mM Isopropyl â-D-thiogalactopyranoside (IPTG, Sigma) was used to induce protein expression. The induced *E. coli* cells were harvested and lysed by sonication for 15 min on ice in the GST suspension buffer (20 mM Tris–HCl, pH 7.3, 500 mM NaCl, 1 mM DTT, 1 mM EDTA). After clarification, the lysate was loaded onto a GST affinity column. The column was washed with five column volumes of GST buffer. The CPs were then released from the column by incubating overnight at 4°C with a solution containing 0.5 mM Tris–HCl, 150 mM NaCl, 2.5 mM CaCl_2_, 0.1% β-mercaptoethanol and 0.5 U Thrombin protease (Cat# T-6884, Sigma), and then eluted from the column with 10 column volumes of elution buffer (20 mM Tris–HCl, pH 7.3, 500 mM NaCl, 10 mM DTT, 1 mM EDTA). The fractions of interest were pooled and purified through ion exchange chromatography and gel filtration chromatography (Bio-Rad). The purified proteins were detected by Western blotting with a BBSV CP antibody.

### RNA binding assay

The *E. coli*-expressed, purified WT and mutated CPs were separated on a 15% SDS-PAGE gel, and transferred onto a piece of nitrocellulose membrane. The similarly expressed green fluorescent protein (GFP) was used as a negative control. A non-radioactive North-western blot procedure was used to assess the RNA-binding activities of CPs bound on the membrane [4], using *in vitro* transcribed digoxigenin-11-UTP-labelled BBSV RNA as the probe. The binding reaction was allowed to proceed for 2 h in the binding buffer containing 10 mM Tris–HCl, pH 7.5, 1 mM EDTA, 100 mM NaCl, 0.05% Triton X-100, and 1X Denhardt’s reagent. The bound RNA was then detected using the anti-digoxigen conjugated Fab-alkaline phosphatase (1:3,000 dilution, Roche), and the substrate solution containing nitroblue tetrazolium (NBT) and 5-bromo-4-chloro-3-indolylphosphate (BCIP, Sigma).

Any experimental research that is reported in our manuscript was performed with the approval from Ethics Committee of China Agricultural University. No any experimental research on humans and animals in our manuscript.

## Competing interests

The authors declare that they have no competing interests.

## Authors’ contributions

X Zhang, D Li, C Han, and J Yu conceived the study. X Zhang and X Zhao carried out the experiments. X Zhang, F Qu, and D Li wrote the manuscript. Y Zhang, S Niu and Y Zhang provided various reagents and valuable advices throughout the study. All authors read and approved the final manuscript.
